# The Cross-Cultural Validity of Post-Traumatic Stress Disorder and Post-Traumatic Stress Symptoms in the Indian Context: A Systematic Search and Review

**DOI:** 10.3389/fpsyt.2019.00439

**Published:** 2019-07-04

**Authors:** Andrew Roderick Gilmoor, Adithy Adithy, Barbara Regeer

**Affiliations:** ^1^Athena Institute for Research on Innovation and Communication in Health and Life Sciences, VU University Amsterdam, Amsterdam, Netherlands; ^2^Private Practitioner, Pune, India

**Keywords:** post-traumatic stress disorder, PTSD, epidemiology, global mental health, transcultural psychiatry, India

## Abstract

**Background:** The cross-cultural validity of the construct of post-traumatic stress disorder (PTSD) has been a life-long debate in the field of trauma. Its validation in a setting such as India—a nation prone to considerably traumatic events such as conflict, natural disasters, and sexual violence against women—warrants exploration.

**Objective:** To describe how PTSD and post-traumatic stress symptoms (PTSS) are conceptualized in the Indian context by systematically examining the evidence of studies that investigate PTSD and PTSS in India.

**Methods:** A systematic search in PubMed, Web of Science, and Science Direct yielded a total of 56 studies that discussed one or multiple aspects of PTSD and PTSS in India. Data relating to types of events, populations, diagnostic tools, manifestations, and interventions were extracted and analyzed.

**Results: **Eleven of 29 Indian states and 2/7 union territories were represented in the 56 included studies, with most studies (n = 21) originating from Tamil Nadu. Natural Disasters (n = 28), War/Conflict (n = 10), and Medical conditions (n = 7) were the top three most commonly investigated traumatic events. The majority of studies focused on entire communities (n = 16), while children and adolescents made up the second largest group (n = 14). Less attention was paid explicitly to male (n = 3) or female (n = 4) victims. Twenty-five different methods for screening for PTSD were identified, with the most common being the impact of events scale (n = 14). The majority of studies reported the screening and clinical diagnosis of PTSD by professional health care providers (n = 24). Abuse scored the highest average prevalence of PTSD at 52.3%, while the lowest was 16.4% due to man-made accidents. Overall, there was a lack of assessment on trauma-specific interventions, though psychosocial support was the most commonly mentioned intervention.

**Conclusions:** Results indicate diversity in approaches for identifying, measuring, and treating PTSD and PTSS in the Indian population and how sociocultural norms influence its manifestation in this population. Future research calls for the development of culturally sensitive approaches to identifying and addressing PTSD and PTSS in India.

## Introduction

Post-traumatic stress disorder (PTSD) is among the most controversial disorders to be described by the *Diagnostic and Statistical Manual of Mental Disorders* (DSM), with regards to the boundaries of its classification, its diagnostic criteria, its main assumptions, and its clinical implications (1). Generally speaking, it is described as the persisting condition that follows after direct or indirect exposure to a traumatic event. According to the DSM-5 criteria for PTSD, this condition consists of a number of criteria, namely, post-traumatic stress symptoms (PTSS) of intrusion or re-experiencing the event (Criterion B), avoidant symptoms (Criterion C), negative alterations in cognitions and mood (Criterion D), and increased arousal symptoms (Criterion E) (2).

From a relativist’s perspective, where the notion holds that all human behaviors are culturally patterned, the cross-cultural validity of the concept of PTSD has been questioned for many years (3–5). As it stands, the application and categorization of a PTSD diagnosis is based on agreed upon notions of how a person is supposed to react to traumatic events. These agreed upon notions, and therefore manifestations of PTSD, are in turn shaped by cultural norms, coping strategies, and availability of support (3). These subjective influences on the experiences of trauma pose a number of challenges in addressing PTSD in differing sociocultural contexts.

Firstly, how traumatic events themselves are defined and understood may differ in different sociocultural contexts. As Swartz (6) expresses, the intention behind labeling an event as traumatic is to convey that the experience is beyond what might be considered a normal human experience (4, 6). When it was first conceptualized—as “Nostalgia” in the mid-1700s and the more commonly known term “Shell Shock” in the early 1900s—PTSD-like symptoms were most commonly associated with war veterans returning from combat (7). However, in the present day, what may seem out of the realm of normal experience in one culture may possibly be considered normal in others. It may be questionable, for instance, whether violence due to conflict can be considered a traumatic event in an environment where conflict is the norm.

The second dilemma comes with the diagnosis of PTSD, as symptoms may differ with different experiences as well as within difference contexts. In the DSM-IV criteria for PTSD, “feelings of intense fear, helplessness or horror” were made explicit criteria for a PTSD diagnosis (8). This criterion was later subjected to criticism as these responses were not necessarily relevant in responses to other traumatic events, such as sexual abuse, where feelings of guilt and shame were more commonly evident (9). Additionally, responses to traumatic events were seen to be culturally subjective (1). Studies, such as those by Rajkumar, where an absence of functional impairment and avoidance was observed in victims of the 2004 Boxing Day tsunami, call to question the nosological validity of PTSD in non-western countries due to the apparent absence of certain symptoms in some contexts and their expression in others (5).

The complexity of PTSD is further exemplified when considering the different classifications of traumatic events, time of exposure, and their subsequent impact. A distinction is often made between interpersonal (e.g., sexual violence and physical abuse) and non-interpersonal traumas (e.g., natural disasters and road accidents), and their consequences. Several studies report higher rates of PTSD as well as more severe PTSD symptoms in victims of interpersonal trauma compared to non-interpersonal trauma (10–12).

An additional distinction is made, by some, between big T and small t traumas. Big T traumas are the kind of events mentioned in criterion A of PTSD in DSM-5—“exposure to actual or threatened death, serious injury, or sexual violence” (2). Such events include earthquakes, terrorist attacks, rape, or tsunamis. Small t traumas are more common events such as humiliation, or emotional neglect, yet have had a lasting negative effect on the self or the psyche (13).

Repeated exposure to trauma in childhood severely exacerbates the symptoms of PTSD—often referred to as complex PTSD (C-PTSD) (14, 15). Since young children have more of a sense of internal locus of control (e.g., Father beats me because I am bad), feelings of intense guilt and shame are common (16). Irrational beliefs, difficult emotions, as well as disturbing or numbing body sensations that occur in the developing time of a child, as a consequence of such traumatic events, have the potential to perpetuate themselves into adult life (15).

India—with its 1.3 billion population, is a subcontinent extremely rich in both physical and cultural diversity. Its roughly 3 million square kilometers is split into 29 states—each constituting their own unique natural environments, languages, political structures, and social and economic constructs. In spite of this wealth in diversity, unfortunately, also bids abundance in disparity and exposure to trauma. From major earth quakes in the western state of Gujarat, to tsunamis and cyclones in the eastern states of Tamil Nadu and Odisha, to political conflict in the northern Kashmir, and an ever-rising income inequality among society, India has seen its share of traumatic events in the current millennium. While Indian mental health services have undergone exceptional development since the country’s independence nearly seven decades ago, there remains a disparity between the large population of individuals suffering from mental illnesses such as PTSD and those who have access to available services (17).

The cross-cultural validation of PTSD in a setting such as India is paramount to improved mental health care. One major importance of ensuring cultural validity is to facilitate communication between health professionals and the community (4). The mismatch between mental health professional and local perspectives of what defines PTSD may lead to less effective diagnostic and treatment practices, further contributing to the unmet need for mental health care services, particularly in lower-middle income countries (LMICs).

Previously published literature has provided a concise overview of studies that explore PTSD in the Indian context. In their 2016 study, Pillai et al. open the discussion on the characteristics and cultural validity of PTSD in India, touching upon a variety of selected studies on the subject (18). To our knowledge, however, there has been no systematic search that covers a larger scope and analysis that offers more in-depth insight on how PTSD and PTSS are studied and conceptualized in Indian settings, nor has there been a critical review of the studies included. In this review, we wish to understand what language of distress is used to describe PTSD and PTSS in research, clinical, as well as community settings in India. Therefore, the aim of this research is to describe how PTSD is conceptualized in the Indian context by systematically examining the evidence of studies that investigate PTSD and PTSS in India.

## Methods

### Study Design

In order to obtain the most reliable and comprehensive evidence that describes PTSD and PTSS in the Indian context, a systematic search and review was undertaken. This approach was chosen because while it offers the advantages of a comprehensive search process of those conducted in a systematic review, it also incorporates multiple study types and is not limited to randomized control trials, allowing for a broader and more accurate picture of the conceptualization of PTSD in India (19).

### Data Search Strategy

Three major databases (PubMed, Science Direct, and Web of Science) were screened to identify journal publications relating to psychological trauma and PTSD in India. The search strategy included terms such as “Trauma” OR “Post Traumatic Stress Disorder” OR “PTSD” AND “India.” Studies were screened on a number of pre-determined inclusion and exclusion criteria. Potentially eligible studies included primary data sets published in peer-reviewed scientific journals, studies that were conducted in India, and studies that concern the topic of psychological trauma and PTSD and were written in English. Bibliographies of secondary data sets were also searched for publications of primary research found to be relevant in the scope of the review. Due to the specificity of the topic, no date restrictions were applied, thus included all studies published up until time of extraction, March 2017. Posters, abstracts, editorials, commentaries, reports, and studies with a focus on mental health disorders that do not include PTSD were excluded from the search.

**Figure 1:**
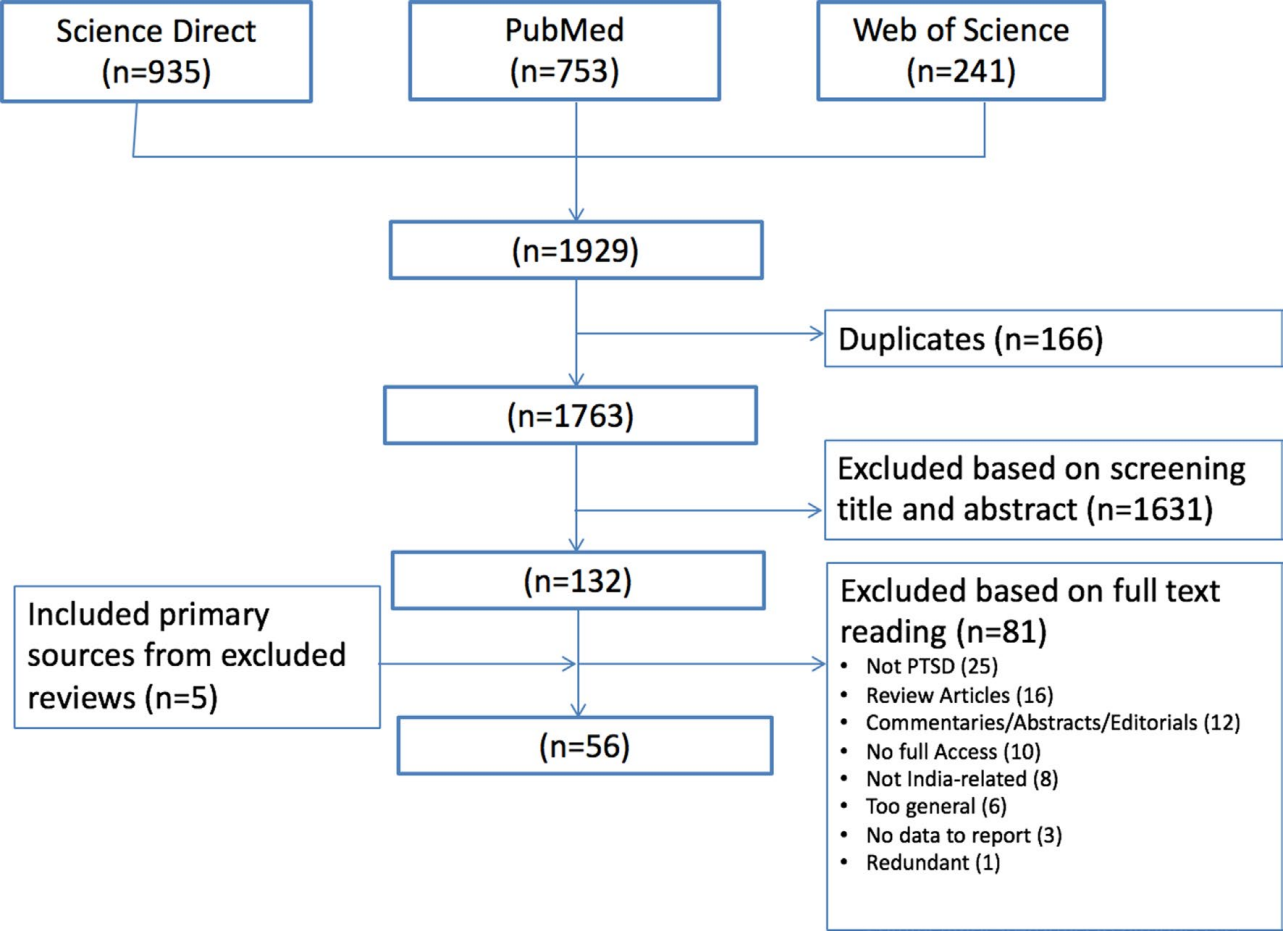
Flow chart overview of article selection process.

Based on the selection criteria above, a total of 1,763 titles were identified from the selected databases. Author AG then read all titles and abstracts and excluded those that did not meet the inclusion criteria. The author then read the full text of the remaining 132 studies. An additional five articles retrieved from selected reviews were also extracted. Finally, after reading all articles, a consensus was made by authors AG and AA on the final selection of 56 studies, for which all selection criteria were met. **Figure 1** provides a full overview of the selection process.

### Data Extraction

For quality assurance, the first 10 extractions were done by both authors AG and AA. After the quality of data extraction was assured, author AG proceeded to extract the information relevant for this review from the 56 selected studies. The type of information retrieved from these studies included the following: types of traumatic events being studied, the location of the study, details of the population being studied, the description used to describe PTSD, what diagnostic tools were used to identify PTSD and by whom, the prevalence of PTSD or PTSS identified, risk factors for PTSD, what interventions were applied if applicable, what challenges in care or gaps in the research were mentioned by the authors, and what recommendations could be made based on the studies.

### Data Analysis

For analysis purposes, the data provided in an excel sheet were sorted according to region and type of traumatic event studied. The full extraction table is in the appendix of this manuscript. Descriptive statistics were used to identify the most commonly reported extraction units: types of traumatic events, regions of study, diagnostic tools utilized, and PTSD or PTSS prevalence.

### Methodological Assessment

Using a Standard Quality Assessment Criteria for Evaluating Primary and Secondary Research Papers, the selected studies were assessed for their methodological quality. This particular assessment criterion, designed and developed by the Alberta Heritage Foundation for Medical Research (AHFMR), was selected, as it is one of the most commonly used criteria for methodological assessment of scientific research (20). Two different scoring systems—one for the assessment of qualitative research reports and one for the assessment of quantitative research reports—were utilized for the methodological assessment. Articles were rated a score between 0 and 1, where a rating of 0.8 and above was considered excellent, a rating of 0.79–0.6 was considered satisfactory, and a rating of 0.59 and below was considered unsatisfactory. For quality assurance, the first 14 studies were scored independently by authors AG and AA. Once the scoring standard was agreed upon, author AG then completed the assessments for the remaining articles. Studies with an unsatisfactory score were still included in the final analysis, however with acknowledgement of their below-standard rating.

## Results

### Study Selection and Characteristics

The 56 studies included in this review included a range of different study designs. Most of the extracted studies consisted of health assessments (n = 26), in which the investigators assessed the magnitude of psychiatric morbidity following traumatic experiences in particular settings. Thirteen studies were prevalence studies in which investigators specifically measured the prevalence of PTSD or PTSS following traumatic experiences using various screening measures. Eight studies were intervention studies that investigated the impact of specific interventions on the prevalence of PTSD and PTSS. Four case studies that focused on specific cases of PTSD and PTSS were also included. Two risk assessment studies exploring the risk factors for PTSD in specific settings, and two studies focusing on the development and validation of PTSD screening tools were also included. Only 4 out of the 56 studies were qualitative in nature (21–24), in which qualitative research approaches were employed to unravel the experiences of trauma and their consequences from survivors’ perspectives. Lastly, one needs assessment study was also included in the review.

The methodological quality of the included studies in this review also widely vary. Based on their methodological quality rating, 31 studies scored an excellent, 17 studies scored satisfactory, and 6 studies scored unsatisfactory. These six studies were still included in this review, despite their unsatisfactory rating due to their contribution to an already limited number of studies in illustrating the scope of trauma in India. Two studies did not undergo methodological assessment, as these were intervention studies that were purely descriptive in terms of processes. **Table 1** ****provides an overview of the assessment for methodological quality of the selected studies.

**Table 1:** Study details of included articles.

Author	Study design	Location of study	Type of event studied	Sample population and size	Tool applied	Prevalence/magnitude of PTSD or PTSS	Methodological score
**Abuse**							
Bhaskaran et al. (25)	Case study analysis	Tamil Nadu	Sexual abuse	2 children, aged 2 and 3	Not specified	PTSD symptoms of avoidance, intrusion and alteration in arousal found in both case studies (100%)	0.68
Bhaskaran et al. (26)	Health assessment	Karnataka	Sexual abuse	40 children and adolescents	Children’s Global Assessment Scale. Administered by: not specified. Time after trauma: not specified.	20% PTSD, 35% depressive disorder, 17.5% adjustment disorder, 12.5% dissociative disorder	0.9
Tichy et al. (27)	Health assessment	Tamil Nadu	Domestic abuse	64 women	Post-traumatic stress diagnostic scale. Self-reported. Time administered after event is not specified.	37% of participants suffered from chronic PTSD, while 25% suffered from acute PTSD (or had indications of clinically significant PTSD). Another 22% were indicated to have clinically significant acute stress disorder. Duration of PTSD symptoms was solely predicted by the belief that domestic abuse was a societal problem. Distress was predicted by one’s ability to correctly recognize one’s own experience as abuse.	0.91
**Exposure to violence**							
Bhatia et al. (28)	Prevalence study	Delhi	Stampede	32 adolescent girls	Child’s reaction to traumatic events scale-revised (CRTES-Revised). A score of 28 or higher is recommended for a PTSD diagnosis. Administered directly after, then at 8 weeks, then at 6 months. Administered by psychiatrists.	22 children had symptoms of PTSD, 5 had moderate distress. 11 comorbid depressive disorder, 6 comorbid phobic disorder and generalized anxiety disorder. Two developed full-blown PTSD after 6 weeks. There was a significant decrease in CRTSEQ-R scores after 6 months.	0.75
Cheng et al. (29)	Health assessment	New Delhi	Vulnerable environments	500 vulnerable adolescents aged between 15 and 19 years old	Six items of the PTSD Checklist-civilian version. A score of 13 or higher indicated PTSD. Administered by: not specified. Time after trauma: not specified.	Of all the study sights, new Delhi had one of the lowest scores of PTSD, with a prevalence of 16.3%. Lowest percentage of depression and suicidal thoughts was also found in Delhi at 13% and some 13.9%, respectively. Mental health problems seem to be significantly associated with family support and neighborhood connection.	0.86
Raju (30)	Case study analysis	Maharashtra	Witness to violence	1 adult male witness to violence	Psychiatric evaluation/not specified. Administered by trained psychiatrist, 35 years after event.	not applicable	0.81
**Man-made accidents**							
Kulkarni et al. (31)	Prevalence study	Maharashtra	Man-made accident	47 relatives of patients in ICU	Impact of events scale-revised, administered between 48h and 72h after event. Administered by medical students.	Post-traumatic stress response identified in 23% (n = 11) of Indian respondents. Median IES total score = 24 (total score of above 30 used to identify relatives with significant PTSS)	0.86
Seethalakshmi et al. (32)	Health assessment	Maharashtra	Motor vehicle accident	30 patient vehicle crash victims	Impact of Events Scale, where a score greater than 26 indicates presence of PTSD symptoms, and a score higher than 40 indicates PTSD. Assessed by qualified psychiatrists 1–6 months after incident.	30% (n = 9) exhibited PTSD symptoms, while 20% (n = 6) had scores higher than 40, suggesting PTSD. Female gender was associated with higher IES scores. There was a significant correlation between negative emotions and other symptoms, except for avoidance. High prevalence of anxiety and depressive symptoms found (57%).	0.86
Sethi and Bhargava (33)	Health assessment	Haryana	Suicide	24 children aged between 6 and 16 with a family member who committed suicide	Childhood post-traumatic stress reaction index (CPTSRI). Not specified who administered. Administered 6–24 months after the event.	21% (n = 5) children met the criteria for PTSD (2 male and 3 female). Almost half of the children met the criteria for major depressive disorder. All 3 children who discovered the body of the victim had PTSD and major depressive disorder. 0.04% panic disorder, 0.13% conduct disorder	0.75
**Medical condition**							
Kumar et al. (34)	Prevalence study	Karnataka	Medical condition	152 postpartum mothers	Mini international neuropsychiatry interview schedule administered within 3–8 days of event.	No PTSD found. 26% depressive disorder. Others included: obsessive harm to child, social phobia, and panic disorder	0.85
Margoob et al. (35)	Health assessment	Jammu and Kashmir	Medical operation	8 men and 8 women accidental or therapeutic amputees	Clinical administered PTSD scale (CAPS). Administered by: psychiatrist. Duration after: not specified.	80% (N = 10) individuals who lost their limb in a traumatic accident met criteria for PTSD. two had elevated scores on CAPS but did not meet the threshold. PTSD cluster symptoms were related to the traumatic events which lead to the loss of the body part. 16.7% (n = 1 out of 6) met criteria for PTSD of those who had undergone therapeutic surgical amputation.	0.5
Mohanti and Kaur (36)	Prevalence study	Delhi	Medical condition	31 adult cancer survivors	Mini international neuropsychiatry interview schedule administered by physicians. Duration after not specified.	Social anxiety and PTSD was elicited in 19% of the patients (no differentiation between the two). 13% reported depression.	0.6
Prashanth et al. (37)	Health assessment	Karnataka	Physical injury	378 patients suffering facial and non-facial injuries	Impact of events scale, with a score of 35 and above being a probable PTSD diagnosis. Tested at date of discharge, 1st month and 6th month post-operative.	Patients with facial disfiguring injuries scored significantly higher on IES and HADS compared to extremity injury and non-disfiguring facial injury. Actual PTSD prevalence not provided	0.82
Prashanth et al. (38)	Health assessment	Karnataka	Physical injury	460 patients suffering facial and non-facial injuries	Impact of events scale, with a score of 35 and above being a probable PTSD diagnosis. Tested at date of discharge, 1st month and 6th month post-operative.	Patients with facial disfiguring injuries scored significantly higher on IES compared to non-disfiguring facial injury, and in addition, female patients were more likely to get PTSD	0.96
Srivastava et al. (39)	Health assessment	Maharashtra	Medical condition	50 male amputees	Trauma symptom inventory: 100 item test of post-traumatic stress and other psychological disorders. T scores higher than 65 are considered significant. Cut off is taken at 70 for validity indicators. Administered by psychiatrist.	Only marginal elevation is noted on scales of defensive avoidance, dissociation, intrusive experiences and depression.	0.68
Tavane et al. (40)	Tool development	Karnataka	Medical condition	96 adult patients suffering facial trauma	Kannada Version of Impact of Events Scale	The kannada version of the IES had satisfactory reliability, internal validity, and test-retest reliability	0.89
**Natural disasters**							
Becker (41)	Intervention study	Tamil Nadu	Tsunami	100 women	Impact of event scale. Time after trauma: 2 years.	At baseline: 32% reported severe symptoms. Post intervention: 22% reported moderate symptoms	0.61
Bhushan and Kumar (42)	Health assessment	Tamil Nadu	Tsunami	130 tsunami adolescent victims ranging between 10 and 16 years old	Impact of event scale administered 1 year after the tsunami as well as the children’s revised impact of events scale, version 13 (CRIES-13).	Children scored high on post-traumatic stress. Intrusion was significantly correlated with avoidance. Both were significantly correlated with total IES score. Female gender correlated with a high IES score. Family type played a significant role in IES impact, proving the positive effect of family support system.	0.75
Bhushan and Kumar (43)	Health assessment	Tamil Nadu	Tsunami	20 adult relief workers	Impact of event scale. Administered 4 years after event. Cutoff values: not specified	50% of the relief workers scored above the mean total trauma score. 40% scored above the mean intrusion and avoidance scores. Total post traumatic growth and proactive coping scores of 60% were above the respective mean scores.	0.87
Chadda et al. (44)	Prevalence study	Jammu, Kashmir	Earthquake	450 community members	ICD-10 criteria clinical assessment carried out by a psychiatrist. Administered 5 weeks after the event.	3.3% (n = 10) were diagnosed with PTSD. Though PTSD-like symptoms were reported by more than 2/3rds of the patients. Adjustment disorders, other stress disorders and depressive episodes were the most common psychiatric illnesses diagnosed (at more than 80%). 39.6% Adjustment disorder. 22.6% depressive disorder	0.64
Crabtree (21)	Health assessment	Bihar	Floods	community	Semi-structured interview with questions relating to PTSD. 18 months post flood. Administered by: not specified.	18 months post flood gave onset to symptoms related to PTSD (primarily re-experiencing).	0.6
Descilo et al. (45)	Intervention study	Tamil Nadu	Tsunami	183 adult tsunami survivors	PTSD Check List (PCL-17). Administered 8 months after the tsunami. Cutoff value 50. Administered by: not specified. First administered 8 months after disaster.	Of the 350 subjects recruited, 240 (68%) met the inclusion criteria for PTSD. Effects of intervention were significant after 6 weeks.	0.76
Exenberger et al. (46)	Prevalence study	Tamil Nadu	Tsunami	177 children	Children’s revised impact of event scale-8 (CRIES-8) Measure symptoms of intrusion and avoidance. Administered by trained university students, 4 years after the event.	CRIES-8: children had more avoidance symptoms than intrusion. Only one child scored above the cutoff for a PTSD diagnosis. There was an association between post-traumatic growth with post-traumatic stress symptoms and age.	0.77
Jeyanth and Jawahar (47)	Intervention study	Tamil Nadu	Tsunami	not specified	not specified	not specified	N/A
Baddam John et al. (48)	Prevalence study	Tamil Nadu	Tsunami	523 children and adolescents	Child behaviour check-list post-traumatic stress disorder scale Tamil revised (CBCL-PTSD-TR). Post graduate students performed the assessment after a 5-day training. Administered 2 and 5 months after tsunami	65% experienced loss of property, while 9% loss of life, and 2.2% loss of both. At 2 months: 70.7% (n = 355) had PTSD with a mean IES score of 26. PTSD higher in girls than boys (75.9% girls, 62.8% boys), and higher in older age groups. At 6 months: 55 of the 147 participants who were free of PTSD during 1st assessment had developed PTSD—indicating delayed onset. Overall prevalence is 81.6%	0.85
Kar and Bastia (49)	Prevalence study	Orissa	Cyclone	108 secondary school adolescents	Mini international neuropsychiatry interview schedule for children and adolescents (MINI-KID). Administered by a clinical psychiatrist, 14 months after cyclone.	26.9% PTSD, 17.6% major depressive disorder, and 12% generalized anxiety disorder. 37.9% of sample population had some kind of diagnosis. Comorbidity was found in 39% of adolescents with a psychiatric diagnosis. Adolescents from middle socio economic status were affected the most.	0.59
Kar et al. (50)	Prevalence study	Tamil Nadu	Tsunami	353 directly exposed and 313 indirectly exposed adults	Self-rating scale for PTSD: 17 item scale which corresponds closely to DSM-III criteria. Conducted by college students, 4 years after the event.	SRQ positive: 84.4%, 70% and 77.6% in direct, indirect and total respectively. PTSD prevalence of 72%, 69.6% and 70.9% in direct, indirect and total, respectively. Depression 33.6%, anxiety 23.1%, co morbidity 44.7%	0.96
Math et al. (51)	Prevalence study	Andaman and Nicobar Islands	Tsunami	12,784 displaced and non-displaced tsunami survivors	ICD-10 criteria clinical assessment by trained psychiatrist within the first 3 months following the disaster	Of the 475 survivors who had at least one psychiatric diagnosis, 53 had a diagnosis of PTSD (11.2%). No significant difference between displaced and non-displaced groups. 0.4% prevalence in total. Depression 21.5%, panic disorder 12%, anxiety disorder 5.5%	0.85
Math et al. (52)	Health assessment	Andaman and Nicobar Islands	Tsunami	535 primary, secondary and tertiary child and adolescent survivors	ICD-10 criteria clinical assessment by trained psychiatrist. During first 3 months.	PTSD diagnosed in 10.8% (n = 4) primary and secondary survivors. Adjustment disorder 13.5%, depression 13.5%, panic disorder 10.8%, schizophrenia 1%	0.4
Pyari et al. (53)	Risk assessment	Tamil Nadu	Tsunami	485 tsunami community survivors	Impact of events scale-revised. 6 months after the disaster. Trained interviewers administered the scales.	Prevalence not investigated. But odds ratio for risk of PTSD indicated that women were 6.35 times more likely to get PTSD than men. Other risk factors include: older age, living in area of highest destruction, death of close relatives, injury to self or family. A lower standard of living correlated with a higher risk of PTSD. Protective mechanisms against PTSD were satisfaction of services received, frequency of counselling received (3x or higher), and absence of fear of reoccurrence	1
Rajkumar et al. (5)	Health assessment	Tamil Nadu	Tsunami	567 tsunami community survivors	Impact of events scale-revised and Complicated Grief Assessment scale (CGA) which assesses the traumatic grief reactions within the previous month. Administered 9 months after disaster, by a team of psychiatrists and trained medical staff.	15.1% of respondents were diagnosed with post-traumatic stress symptoms. Participants scored significantly less on the avoidance subscale when compared to the hyperarousal or intrusion subscale. Those with or without PTSS did not differ significantly on functional impairment as was defined by their inability to return to their pre-disaster occupation. Risk factors for PTSS include: presence of traumatic grief, death of children, psychical injury, female gender, men handling corpses during relief work, and reporting more financial losses. Adequate financial aid significantly protected against PTSS.	0.96
Rajkumar et al. (54)	Health assessment	Tamil Nadu	Tsunami	643 tsunami community survivors	Impact of events scale-revised used to diagnose PTSS and PGD. Administered 9 months following disaster, by a team of psychiatrists and trained medical staff.	23.2% (n = 149) diagnosed with PGD, PTSS or both. 34.5% of bereaved survivors diagnosed with PGD, PTSS or both. Prevalence of PTSS without PGD was 8.1%, and 8.8% in bereaved survivors.	1
Roy (55)	Health assessment	Tamil Nadu	Tsunami	Tsunami community survivors	Disaster-related questionnaire (not specified) administered by two hospital paramedics. Time after event not specified.	17% of patients found to exhibit PTSD symptoms (panic attacks, nightmares, insomnia, fear of water, being startled by loud sounds, and palpitations).	0.32
Roy et al. (56)	Health assessment	Gujarat	Earthquake	133 rural displaced victims	A validated 6-item General Health questionnaire administered by one community health worker (items were: repeated images, nightmares, easily startled, anxiety and discomfort, persistent sadness). 2 yearts after earth quake.	PTSD was marked 3–6 months after the event, but was minimal 2 years after the earthquake. Sadness about the event was the only residual PTSD symptom (84%).	0.55
Suar et al. (57)	Health assessment	Orissa	Cyclone	65 affected people	Clinical assessment. Administered by a trained psychologist 3 months post disaster.	89% (n = 58) of affected persons found to have PTSD, compared to 11% (n = 7) in the unaffected controls. Assessment was made after 3 months following the disaster.	0.73
Suar et al. (58)	Health assessment	Tamil Nadu	Tsunami	416 tsunami survivors	Clinical interview using DSM-IV, administered 14 months after the event by trained university undergraduates	Not specified, however results indicate that indirect effects of tsunami exposure on trauma* via *the loss of resources were a more potent predictor than the direct effects of exposure on trauma	0.96
Telles et al. (59)	Risk assessment	Bihar	Floods	1,289 people directly exposed flood victims	Screening Questionnaire for Disaster Mental Health (SQD), administered 1 month after the disaster. Not specified who administered them.	PTSD prevalence not stated clearly, but results show that being age 60 and older posed a higher risk for PTSD. No sex differences were found in this study.	0.96
Telles et al. (60)	Intervention study	Bihar	Floods	1,089 adult male participant flood survivors, but only 22 participated in intervention	Screening Questionnaire for Disaster Mental Health (SQD). Administered 1 month after the floods. And the Visual Analogue Scale – self-rater test to measure fear, anxiety, disturbed sleep, and sadness (indicators of emotional distress/PTSD). Self-rated.	Average pre-intervention score for PTSD were 4.5 on the SQD test. Sadness was the only significant symptom to decrease after the yoga intervention as measured by the VAS.	0.86
Telles et al. (61)	Intervention study	Andaman Islands	Tsunami	47 indigenous and mainland tsunami survivors	Visual Analogue Scale. Administered 1 month after disaster, self-rated.	Fear, anxiety, sadness and disturbed sleep all measured significantly less after intervention	0.64
Varghese (62)	Intervention study	Tamil Nadu, Kerala, Andra Pradesh	Tsunami	In Kerala, 11,831 people were seen. 176 were seen by mental health team	not specified	12 cases of PTSD out of the 176 cases seen by the mental health team in Kerala	N/A
Vijayakumar et al. (63)	Health assessment	Tamil Nadu	Tsunami	230 adolescents	Child post-traumatic stress reaction index (CPTSD RI)	Unclear what prevalence were found. However, it was found that positive family history correlated significantly with affective symptoms, hyperactivity, somatic symptoms and symptoms related to PTSD	0.91
Vijayakumar et al. (64)	Intervention study	Tamil Nadu	Tsunami	65 adolescent intervention participants and 70 controls	Child post-traumatic stress reaction index (CPTSD RI). Administered 1 year after tsunami by two psychologists and three trained volunteers.	Only hyperactivity problems were significantly reduced after the intervention. Majority of children are likely to be resilient and only children with pre-existing vulnerability require specific and specialized interventions	0.64
Vijayakumar and Kumar (65)	Intervention study	Tamil Nadu	Tsunami	102 adults	Scale developed for PTSD as according to ICD-10 and administered by 2 trained psychologists, 1 year after.	Significant decrease in PTSD scores in intervention group, however no prevalence indicated	0.93
Viswanath et al. (66)	Health assessment	Andaman Islands	Tsunami	475 patients accessing mental health services	Diagnosis made using ICD-10 by qualified psychiatrists. During initial 3 months following disaster.	Overall, 37% adjustment disorder, 11.2% PTSD, 21.5% depression, 12% panic disorder, 5% anxiety disorder. 13% of males (n = 24) vs. 10% females (n = 29) were diagnosed with PTSD. These diagnoses were in the top 3 of diagnoses after adjustment disorder (33% = m, 40% = f) and depression (17% = m, 24% = f). PTSD was higher in displaced women (12%) compared to non-displaced women (9%). No significance in gender differences was found, nor for displacement.	0.82
**Other/non-specified**							
Jadhav and Barua (23)	Case study analysis	Assam	Elephant attack	4 community case studies	Clinical assessment by trained psychiatrist. Time of first evaluation not specified, and last evaluation up to 4 months post event.	1 woman exhibited symptoms of PTSD	0.55
Russell et al. (67)	Health assessment	Tamil Nadu	Not specified	35 adolescents	Impact of event scale with a score of 17 or higher being regarded as a cause for concern’. Self-administered. No specific time after event.	The intrusive symptoms of PTSD were noted more than avoidant symptoms among those adolescents with life events. Adolescents who have psychopathology have significantly different life events. They experienced more parental fighting, increased arguments between parents, and serious illness requiring hospitalization of the adolescent.	0.96
**War/Conflict**							
Thappa et al. (68)	Health assessment	Jammu, Kashmir	Displacement due to war	300 Kashmiri migrant families	Mini International Neuropsychiatry Interview schedule (MINI) Cutoff values: Not specified. Administered by: Not specified. Time after trauma: not specified.	PTSD: 6.83% of migrants had a current PTSD diagnosis versus 2.5% in controls. Generalized anxiety disorder: 13.8%, Major depressive episode: 21.55%	0.86
Bhat and Rangaiah (69)	Prevalence study	Kashmir	Armed conflict	797 young adults ranging from 19 to 24 exposed to armed conflict	PTSD Checklist civilian version (PCL-C). Score ranges from 17 to 85, with a cutoff of 50 and higher as a PTSD diagnosis. Administered by: not specified. Time since trauma: continuous	Out of the sample, 49.81% (n = 397) were classified as having PTSD. Highest proportion of PTSD symptomology occurred in people exposed to 4 events or more. Factors related to conflict exposure that showed a significant association with PTSD were: feeling of living in conflict, family member being killed or missing, being threatened with death, media coverage, and a high level of personal exposure. No gender differences were found.	0.93
Crescenzi et al. (70)	Prevalence study	Himachal Pradesh	Displacement due to war	76 imprisoned and 74 non-imprisoned Tibetan refugees	Harvard Trauma Questionnaire. Administered by two trained lay Tibetan people. Cutoff scores not specified.	20% of imprisoned refugees had a diagnosis of PTSD according to the HTQ. 92% of respondents expressed current thoughts or memories of the most hurtful or terrifying events, 74% difficulty concentrating, 71% sudden emotional or physical reactions when reminded of events, 68% spend time thinking why these events happened to me, 65% feeling irritable or having outbursts of anger, 60% feeling on guard, 52% recurrent nightmares	0.93
Elsass et al. (71)	Needs assessment	Himachal Pradesh	Torture and displacement due to war	102 tortured Tibetan refugees	7 DSM-symptoms of PTSD graded in a 5-point scale. Administered by Tibetan officers. Date since trauma: not specified.	Study does not give an actual diagnosis of PTSD. But an overview of mean % of symptoms experienced: nightmares 2.25%, flashbacks 2.88%, concentration and memory problems 3.03%, restlessness and anxiety 2.61%, feelings of loss and sadness 2.58%, loneliness 2.15%, irritability and anger 2.42%	0.84
George and Jettner (72)	Health assessment	Tamil Nadu	Displacement due to war	50 adult Sri Lankan refugees	Harvard Trauma Questionnaire (only the first part to identify traumatic events) to measure pre-migration traumatic events and the Symptoms Checklist 90R (SCL) - A 90 item checklist that measures psychological distress. Administered by author who is a Social worker/assistant professor. Administered time after event: not specified.	As the number of children increased, psychological distress decreased by 9.685 units. Pre-migration trauma was also a significant predictor of psychological distress, where for each traumatic event experienced, psychological distress increased by 2.707 units. Pre-migration trauma has a stronger influence on psychological distress compared to the number of children. pre-migration trauma also had the strongest impact on daily stressors, followed by psychological distress and host country.	0.91
Hussain and Bhushan (73)	Health assessment	Dharamshala	Displacement due to war	226 Tibetan 1st and 2nd generation refugees	Refugee Trauma Experience Inventory duration after: not specified. administered by: not specified.	Participants scored higher than average on the different scales: traumatic experience - 55.51%, PTS = 67.59%, and PTG = 73.02%. Women reported significantly higher scores compared to men on all factors of traumatic experiences. There were significant generational differences found in all three traumatic experiences. Second generation scored high on survival trauma and deprivation/uncertainty and first generation scored high on ethnic concerns. 1st generation scored significantly higher in IES results.	0.96
Hussain and Bhushan (22)	Tool development	Himachal Pradesh	Displacement due to war	226 Tibetan refugees	Refugee trauma experience inventory	Prevalence was not looked at. But the developed scale had high internal consistency and the factors derived from the scale correlated moderately to strong with the theoretically related constructs of intrusion, avoidance, rumination and post traumatic schema changes.	0.85
Mehta et al. (24)	Case study analysis	Gujarat	Riots	55 women refugees	none	none	0.6
Servan-Schreiber et al. (74)	Health assessment	Dharamshala	Displacement due to war	61 Tibetan refugee adolescents aged between 8 and 17	A newly developed 5 item screening tool. If one item was answered yes, then a clinical interview using DSM-IV criteria was used. Screening was administered at least 12 months after the incident. Screening and psychiatric interviews were done by a psychiatrist and nurse. All psychiatric interviews were reviewed by the psychiatrist.	11.5% confirmed cases of PTSD, 18% suspected cases of PTSD. 11.5% confirmed cases of major depressive disorder, 13.1% suspected cases of MMD. Only two cases confirmed having both PTSD and MMD. No statistical significance in gender differences for either PTSD or MMD.	0.77
Shoib (75)	Prevalence study	Jammu, Kashmir	Not specified (but a region prone to natural disasters and political unrest)	3400 adult subjects	Screening using Life Events Check List, and clinical diagnosis confirmed by psychiatrist using DSM-IV TR and severity of PTSD symptoms assessed using clinical administered PTSD rating scale (CAPS scale). Time after events not specified.	Prevalence of PTSD = 3.76%. Prevalence found to be more in females (2.086 chi square test). Most cases were unmarried, illiterate and belonged to a lower socioeconomic class. Death of a close one comprised the major traumatic event. Onset of PTSD symptoms mostly within 3 months, but also between 3 and 6 months. Symptoms of avoidance and re-experiencing were most prevalent in positive patients (in the 80s%). Symptoms of hyperarousal was the least commonly reported symptom in positive patients (50s-60s%). People in Kashmir have developed a type of resilience perhaps due to the frequent exposure to traumatizing events. This could account for the relatively low prevalence of PTSD found.	0.77

### Synthesized Findings

#### How Is PTSD Defined in the Indian Context?

In this review, 18 out of the 56 studies did not specify a working definition of PTSD that was applied in their investigations. Overall, it was observed that the majority of studies defined PTSD using DSM categorization—highlighting the dominance of western-based definitions of PTSD in the Indian context. No studies were found in which a cross-cultural variation of a PTSD definition was identified. Twenty-two studies in total applied different variants of the DSM classification of PTSD, with the most commonly used definition being that of the DSM-IV (n = 17). Five studies applied the DSM-III original categorization of PTSD, despite the fact that all of these studies were published after subsequent versions were released (30, 35, 50, 70). Interestingly, no studies applied the latest version of the DSM’s criteria for PTSD.

The second most commonly used definition of PTSD applied was that of the 10^th^ version of the International Classification of Diseases (ICD-10) categorization of PTSD (n = 6). Developed by the WHO, it is the standard classification of diseases, endorsed by the World Health Assembly and applied in more than 100 countries worldwide (76). Other studies had more wide-ranging definitions of PTSD that did not fall under either DSM or ICD categorization. For example, in a study investigating emotional distress and PTSD in child-survivors of the 2004 Indian Ocean tsunami, Bhushan and Kumar (42) express with strong intent that describing PTSD as a pathology is a flawed approach. Instead, they describe the symptoms of PTSD as perfectly normal human reactions to very abnormal situations (42). This view is supported by Hussain and Bhushan (73) who studied post-traumatic stress and growth among Tibetan refugees (73).

Two studies, while applying the four major DSM categories of intrusion, avoidance, hyper-arousal, and negative alterations in cognition and mood emphasize the significant distress or impairment in social, occupational, or other areas of functioning as a result of traumatic exposure (21, 45). In a study investigating the effects of yoga on PTSD in tsunami survivors, Descilo et al. (45) emphasized fishermen’s inability to return home and resume their livelihoods as a major characteristic of PTSD.

PTSD is also at times described rather vaguely. In a study on Sri Lankan refugees living in Tamil Nadu, George et al. (72) describe PTSD merely as psychological distress (72). Telles et al. use the term PTSD interchangeably with distress in their 2007 study on the impact of yoga on PTSD in a cohort of 47 community members following the 2004 Indian Ocean tsunami (61). The same study design was applied to a cohort of 22 men following the Bihar floods in 2008 (60). In this study, indicators for emotional distress (fear, anxiety, disturbed sleep, and sadness) were used interchangeably with PTSD. In one study concerning Indian adult cancer survivors, no distinction was made between social anxiety and PTSD (36).

#### What Is the Scope of PTSD in India?

##### Geographical Locations of the Populations Studied

**Figure 2:**
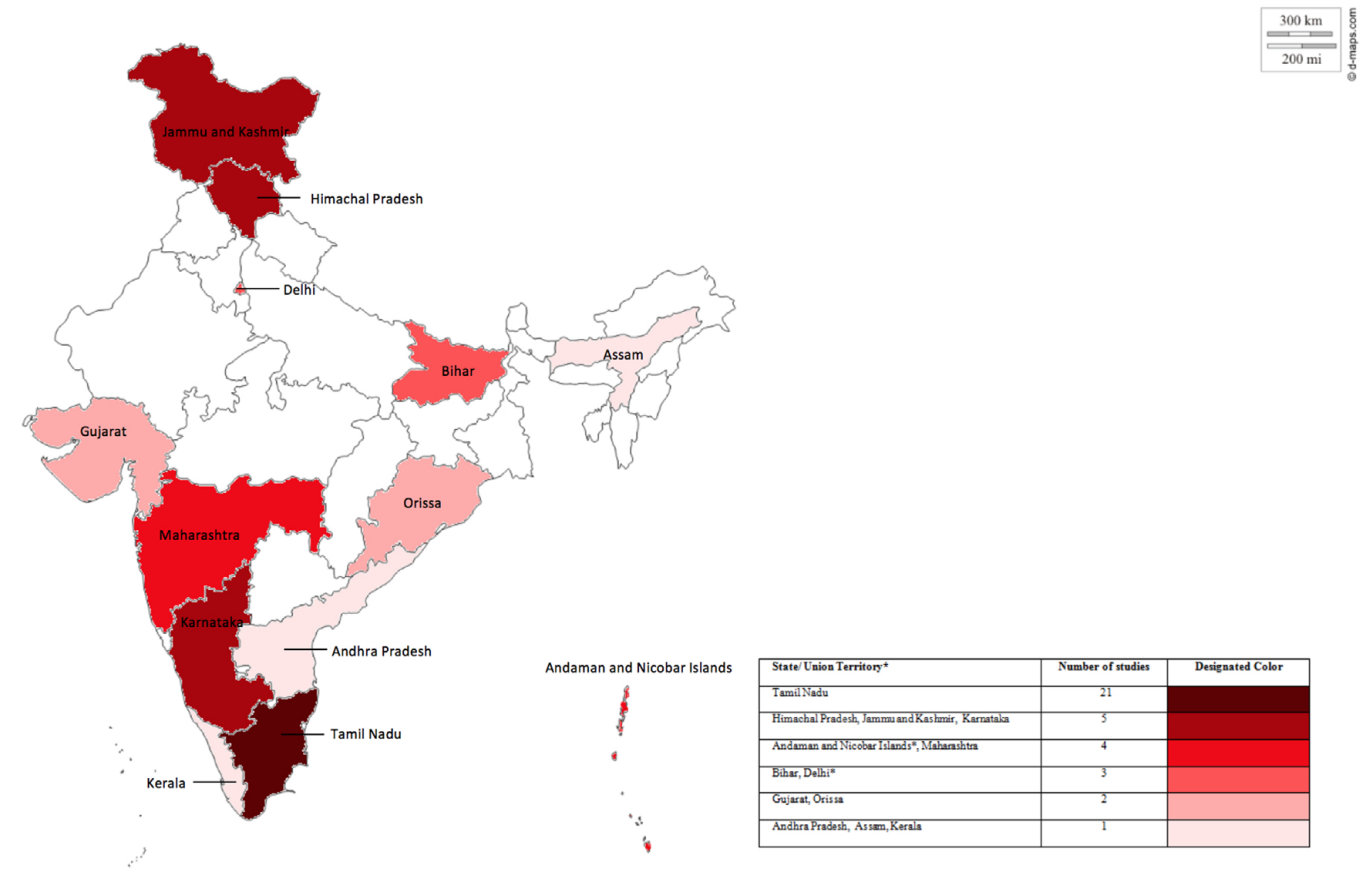
State and union territory coverage, color coded according to number of studies covered per state or union territory. Figure a modification from an original map of India derived from: http://d-maps.com/carte.php?num_car=24855&lang=en.

While study settings were diverse, they covered only a proportion of Indian states (11 out of 29) and union territories (2 out of 7). Most studies cumulatively originated from the south—with Tamil Nadu contributing the highest number (n = 21) due to the 2004 Indian Ocean tsunami. Owing to political unrest and displacement in the north, Jammu and Kashmir (n = 5) and Himachal Pradesh (n = 5) contribute the second highest number of studies reported (**Figure 2**).

##### Types of Events Studied

Among the 56 studies included, 17 different types of traumatic events were investigated. The overwhelming majority of studies saw PTSD as a consequence of exposure to the 2004 tsunami (n = 21), which resulted in 12,405 deaths and displacement of 647,599 persons in India alone (77). Medical conditions such as cancer, facial disfiguration, and limb amputation were the second most commonly reported traumatic experience (n = 7). Displacement due to war was the third most commonly reported traumatic experience (n = 6), owing to the 25-year-long Sri Lankan civil war and subsequent displacement of more than 60,000 refugees to Tamil Nadu in the south and the fleeing of 150,000 Tibetan refugees to Himachal Pradesh in the north.

**Figure 3:**
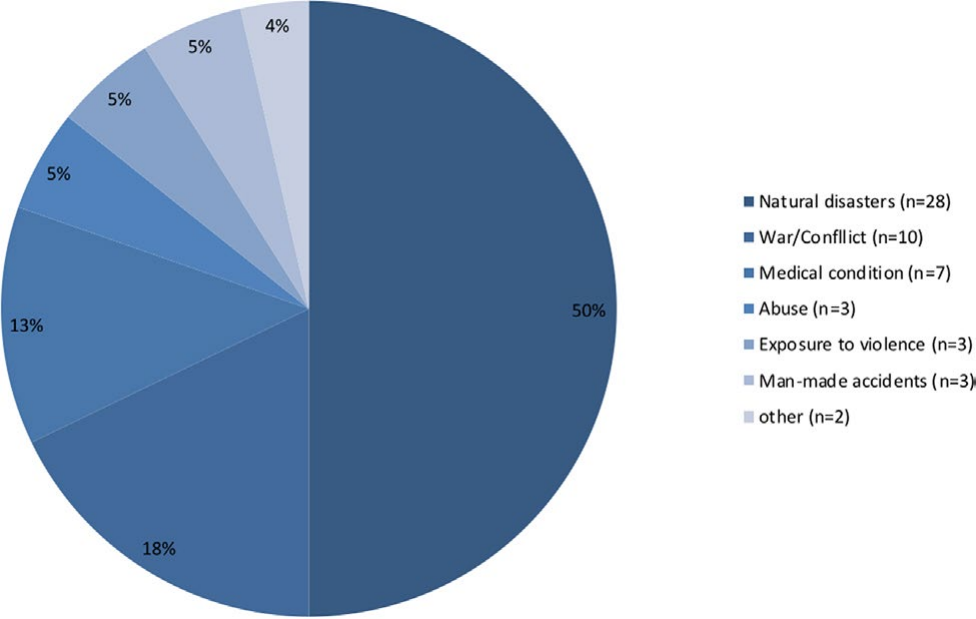
Proportion of traumatic experiences mentioned by the studies, per trauma classification.

When grouped into different classifications, the different types of traumatic events mentioned can be classified as Natural Disasters (n = 28), War/Conflict (n = 10), Man-made disasters (n = 3), Medical conditions (n = 7), Abuse (both physical and sexual) (n = 3), Exposure to violence (n = 3), and other (n = 2) (**Figure 3**).

##### Types of Populations Studied

**Figure 4:**
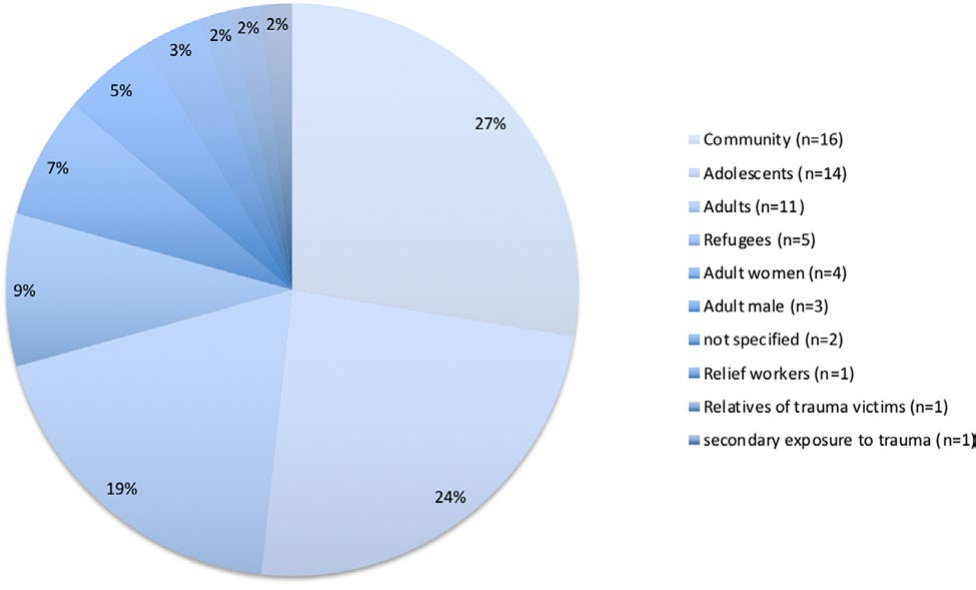
Proportions of different populations studied that were identified.

The different studies included in this review represent a diverse range of populations being studied. The majority of these focused on entire communities (n = 16), without specifying age limitations. Children and adolescents made up the second largest type of population investigated in this data set (n = 14). Adults—non-gender specified—made up the third largest type of population studied (n = 11). There was little difference between the number of studies focusing on male victims and female victims of trauma at n = 3 and n = 4 studies, respectively. Interestingly, few studies focused on populations not directly exposed to traumatic events. One study investigated post-traumatic stress and growth in tsunami relief workers in Tamil Nadu (43). In a cross-sectional prevalence study conducted in a tertiary hospital in India and the U.S., investigators explored PTSD in relatives of patients in intensive care as a result of man-made accidents (31). Another study differentiated between primary, secondary, and tertiary survivors of the Indian Ocean tsunami and compared levels of PTSD in these different groups (52). **Figure 4** below provides a visual representation of the different types of populations studied.

#### What Tools Are Applied for Identifying PTSD in the Indian Setting?

##### Types of Tools

Analysis of the data set revealed a high variation in methods chosen and administration procedure used by the selected studies for screening for PTSD. Among the 56 studies included in this review, 25 different methods for screening for PTSD were identified. The type of method employed for screening for PTSD revealed key differences in perspective held by the authors on the cross-cultural validity of tools used to screen for PTSD.

An overwhelming majority of studies (n = 41) employed already existing screening tools, such as the Impact of Events Scale (IES), the Mini Neuropsychiatry Interview Schedule (MINI), or the PTSD Check List (PCL)—all of which were initially developed and validated in western settings. These screening tools were administered as is with no attempt for adaptation for use in the local context. Few of these studies have provided evidence for their validation in local Indian settings (5, 31, 40, 54, 63–65, 67, 78).

Significantly fewer studies (n = 7) have employed a version of an already existing screening tool for PTSD that they have modified for use in the intended setting. Some of these modifications entail changing the language of the original tools to better fit the sociocultural context of the study population, as was the case for Mohanti and Kaur (36) who adapted the MINI for application in a population of Indian adult cancer survivors (36), and Seethalakshmi et al. (32) who adapted the IES for use in a population of motor vehicle crash victims in India (32). Other modifications entailed either the removal of irrelevant items for a shorter version of the original screening tool, as was the case in the study of Cheng et al. (29), who employed a modified version of the PCL to investigate adolescent vulnerability in LMICs (29) or the use of emic approaches to understand the local definition of key DSM categorizations for PTSD, such as the study conducted by Crabtree (21), who consulted local traditional healers and health workers to establish what the local standard for “functionality” entailed (21).

Even fewer studies (n = 6) developed entirely new trauma inventories or screening tools for PTSD, designed specifically to meet the context and the needs of the population of study. In order to capture the relevant experiences of local Kashmiri residents, Bhat and Rangaiah (69) developed the Exposure to Conflict Checklist, whose items are based on the traumatic experiences reported in previous studies conducted on the Kashmir conflict (69). In another study, Hussain and Bhushan (22) developed the Refugee Trauma Experience Inventory—with its 26-item scale that covers very specific events experienced by Tibetan refugees, falling within the categories of survival trauma, ethnic concerns, and deprivation (22). In an earlier study, Servan-Schreiber et al. (74) developed a screening tool for PTSD, based on local Tibetan child refugees’ perceptions of stress. The most commonly reported symptoms (intrusive memories and nightmares) were used as a screening for PTSD (74).

**Figure 5:**
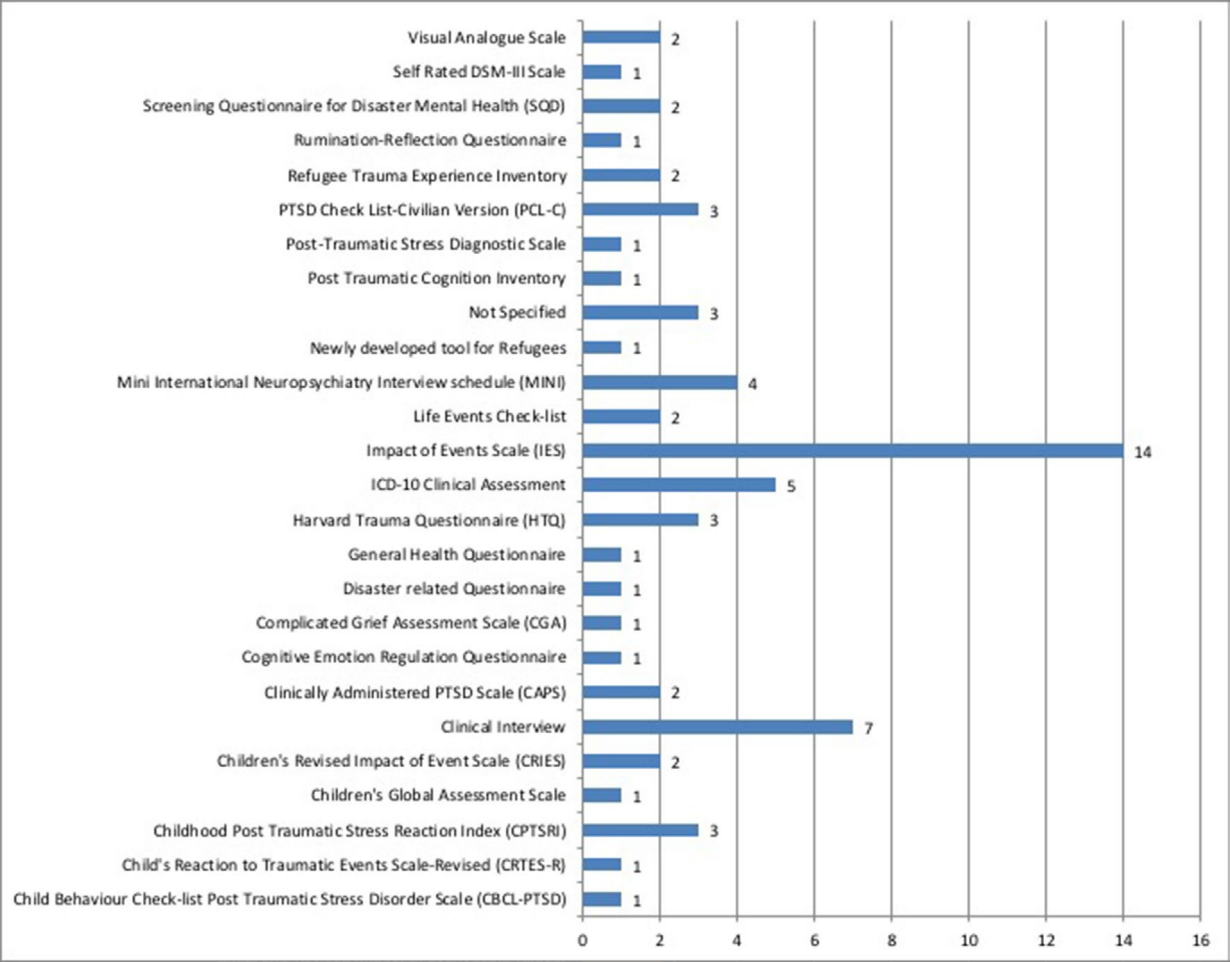
Types of measures for screening and diagnosis applied and the number of studies that used them.

With 14 of the 56 studies reporting its use, the most commonly used screening measure for PTSD in India appears to be various versions of the Impact of Events Scale (IES). Most studies (9 out of 14) applied the original 15-item Likert scale version of the tool, developed in 1979 by Mardi Horowitz (79). Four out of the 14 studies applied the more recent revised version, abbreviated IES-R—a 22-item Likert scale that specifically covers 14 out of the 17 DSM-IV criteria for PTSD (80). One particular study utilized the shorter 8-item version of the scale (48). Non-specified clinical interviews (n = 7) and clinical assessments following ICD-10 criteria (n = 5) were the second and third most frequently used measures for diagnosing PTSD, respectively. Owing to the high attention to PTSD in youth, we identified a relatively high number of scales specifically catered to screening PTSD in children and adolescents. From the data set, five different scales were utilized: The Childhood Post Traumatic Stress Reaction Index (n = 3) being the most common, followed by the Children’s Global Assessment Scale (n = 1), Child’s Reaction to Traumatic Events Scale-Revised (n = 1), Children’s Revised Impact of Event Scale (n = 1), and the Child Behavior Checklist Post Traumatic Stress Disorder Scale (n = 1). **Figure 5** provides an overview of the various measures used to screen and diagnose PTSD in India.

##### Tool Measurement

Several different cutoff values used were identified in different studies that administered the same screening tool for PTSD. In their 2015 study on the psychological impact of facial and extremity injury on patients admitted to various trauma centers in the city of Bangalore, Prashanth et al. applied cutoff scores of 35 or higher using the IES as a probable PTSD diagnosis (37). In a pilot study investigating psychiatric morbidity in motor vehicle crash victims, Seethalakshmi et al. (32) applied cutoff values of 40 and higher for probable PTSD using the same tool. The same study indicated a cutoff value of 26 and higher as a measure for the presence of PTSD symptoms (32). Russell et al. (67) used IES cutoff values of 17 and higher to indicate a “cause for concern” in adolescent patients admitted into primary care facilities in Tamil Nadu (67).

##### Time of Administration

The time point after exposure to a traumatic event at which PTSD is measured also varied widely among the different studies. The time of administration ranged from immediately after the event, as was the case in a study that applied the CRTES-Revised to investigate psychiatric morbidity in adolescent girls caught in a stampede (28), to as late as 35 years following the event, as was the case in a case study investigating the long-term psychological impact on an adult man who witnessed his father being attacked by a group of men as a child (30). The most frequently reported time duration following exposure to a traumatic event was 1 month (n = 6), followed by 6 months (n = 5).

##### Forms of Administration

We identified a wide variation in the different forms of administration of PTSD measures in the data set. An overwhelming majority of studies reported the clinical diagnosis and the administration of PTSD screening measures by professional health care providers (n = 24). Psychiatrists (n = 14) were most commonly reported to carry out the screening or confirm diagnosis. Psychologists (n = 4) and non-physician medical staff (n = 4) were the two second-most common types of personnel to carry out the screening/diagnosis. Physicians (n = 1) and social workers (n = 1) were also noted to administer the measures. Few studies made use of non-professional health care providers to administer PTSD screening measures or to conduct a clinical diagnostic procedure. Lay personnel accounted for four studies, and community health workers accounted for one. Five studies relied on the assistance of trained students for the administration of screening measures, while another five studies relied on self-administration. Sixteen studies did not specify how their PTSD measures were administered.

#### What Is the Magnitude of the Problem of PTSD and PTSS in India?

##### Prevalence of PTSD

**Figure 6:**
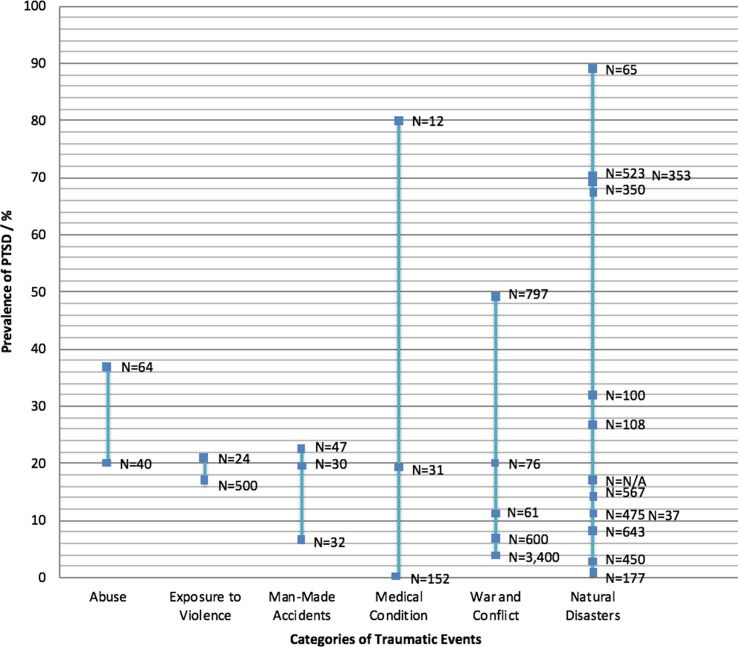
Overview of the different ranges of prevalence measured per category of trauma identified in the different studies measuring PTSD prevalence. Each blue square represents one study. N represents the sample size per study.

Although its definition and criteria vary considerably—as has been illustrated in the previous sections, the findings of this review have also allowed us to shed light on the perceived magnitude of PTSD due to different types of traumatic events across India. Twenty-eight out of the 56 studies measured the prevalence of PTSD in the different populations investigated, where prevalence rates varied widely, both across and within the different trauma categories. The highest average prevalence rate was identified in the category natural disasters, with an average prevalence of 31%. The category natural disasters, however, also had the widest difference in prevalence rates, with the highest prevalence determined to be 89% in a population of survivors assessed three months after the 1999 Orissa cyclone (57) and the lowest prevalence identified as 0.01% in which only one child out of a cohort of 177 child survivors of the 2004 tsunami scored above the cutoff value for PTSD (46). The category abuse scored the second highest in average prevalence at 28%, however with only two studies measuring prevalence rates. The lowest average prevalence of PTSD calculated was 16.4%—attributed to man-made accidents. **Figure 6** below displays an overview of the different ranges of prevalence measured per category of trauma identified in the different studies. This comparison between prevalence rates of PTSD must be interpreted with caution, however, given the differences in sample sizes, time of exposure, and approaches to measure PTSD—factors that influence the outcomes of measure as described earlier.

##### PTSD Symptomatology in India

PTSD symptomatology represents the class of symptoms used within the different studies to determine a PTSD diagnosis or indicate the presence of PTSS. Corroborating with the finding that most studies used western-based DSM categorization for the defining and screening/diagnosing of PTSD, the majority of symptom categories used to describe the expression of PTSD in these populations are also derived from the western DSM classification system, namely, intrusion, avoidance, hyper-arousal, and negative alterations in cognitions and mood (2).

In this review, a number of studies report significantly higher presentation of some symptom categories over others. Four different studies reported significantly lower presentation of the symptom category of avoidance in comparison to the other categories (5, 26, 32, 67). In the study conducted by Russell et al. (67), psychopathology in children attending a primary care adolescent clinic in Tamil Nadu was significantly associated with symptoms of intrusion, whereas those of avoidance were insignificant. In their 2013 study on tsunami survivors also in Tamil Nadu, Rajkumar et al. presented similar findings—participants scored significantly less on the avoidance subscale of the IES-R compared to the intrusion as well as hyperarousal subscales. While the two adolescents presented in the study (25, 26) were clinically diagnosed with PTSD following penetrative sexual abuse, no symptoms of avoidance were reported.

With regards to what the above studies claim about the symptom category avoidance, there are an equal number of studies that report the contrary (42, 43, 46, 75). In the 2012 study investigating psychological morbidity in relief workers for the 2004 tsunami, Bhushan et al. identified that 40% of relief workers scored above average on the intrusion and avoidance subscales of the IES for PTSD. In a psychological assessment of residents in the Jammu and Kashmir conflict region, Shoib et al. (75) identified avoidance and re-experiencing to be the most prevalent symptoms of PTSD found in the population (80%).

Though the majority of studies have used the above-mentioned DSM-derived symptoms to describe the expression of PTSD in the populations studied, few studies have reported PTSD expression in terms of symptoms that go beyond the western-based DSM classification. For example, in addition to the most commonly reported PTSD symptoms of intrusion, avoidance, hyperarousal, and negative alterations in cognition, Roy et al. (55) also explored persistent sadness as a symptom of PTSD when studying the psychosocial impact of the 2000 Gujarat earthquake (55). In a study that explores the impact of yoga on heart rate and PTSSs in a population of Bihar flood victims, Telles et al. (60) use the symptoms anxiety, fear, disturbed sleep, and sadness as indicators for PTSD (60).

##### Risk Factors for PTSD in India

Sixteen studies in this review reported on risk factors for PTSD and PTSS. Among these, the most common risk factor identified in the data set was female gender, with eight different studies reporting a positive correlation between female gender and a positive diagnosis of PTSD (5, 32, 37, 42, 53, 73, 75). Prashanth et al. (37, 38), for example, reported that the mean score for IES was significantly higher in female patients with facial disfiguring injuries compared to male patients at all intervals. In a study specifically exploring gender differences in risk factors for PTSD among tsunami survivors, Pyari et al. (53) concluded that women were 6.35 times more likely to have PTSD than men. The odds of having PTSD were made even higher if the women were married, were above the age of 40, and belonged to a lower socioeconomic status. Pyari et al. ascribe the higher risk of PTSD in women to gender-specific norms and inequalities prevalent in Indian society that make women more disproportionately susceptible to disasters (53). Four studies, however, found no significance between gender and risk of PTSD (59, 66, 69, 74).

Five studies report a correlation with age and risk of PTSD (46, 48, 53, 59, 73). These studies reported that as age increased, participants were significantly more likely to be diagnosed with PTSD. Less commonly reported but also noteworthy risk factors identified were socioeconomic status (49, 53), family size (42), and loss of resources (58).

##### Comorbidities and Other Trauma-Related Disorders

While PTSD was the focus of this review, 15 studies additionally identified and compared the prevalence rates of other trauma-related disorders. The most commonly reported prevalence rates of disorders following exposure to traumatic events other than PTSD include Depression (n = 14), Anxiety Disorder (n = 5), Adjustment Disorder (n = 4), and Panic Disorder (n = 4). **Table 2** provides an overview of prevalence rates for the top four trauma-related disorders presented by studies.

**Table 2:** Overview of prevalence rates for top four trauma-related disorders by study.

Reference	Event	Disorder 1	Disorder 2	Disorder 3	Disorder 4
Bhatia et al. (28)	Stampede	PTSD (69%)	Depressive Disorder (34%)	Phobic Disorder (19%)	Generalized Anxiety (19%)
Thappa et al. (68)	Displacement	Major Depressive Disorder (21.5%)	Generalized Anxiety Disorder (13.8%)	PTSD (6.8%)	
Bhaskaran et al. (26)	Sexual Abuse	Depressive Disorder (35%)	PTSD (20%)	Adjustment Disorder (17.5%)	Dissociative Disorder (12.5%)
Chadda et al. (44)	Earth Quake	Adjustment Disorder (39.6%)	Depressive Disorder (22.6%)	PTSD (3%)	
Cheng et al. (29)	Vulnerable Populations	PTSD (16.3%)	Suicidal Ideation (13.9%)	Depression (13%)	
Kar and Bastia (49)	Cyclone	PTSD (29.9%)	Major Depressive Disorder (17.6%)	Generalized Anxiety (12%)	
Kar et al. (50)	Tsunami	PTSD (70.9%)	Depression (33.6%)	Anxiety (23.1%)	
Kumar et al. (34)	Postpartum	Depression (26%)	PTSD (0%)		
Math et al. (52)	Tsunami	Adjustment Disorder (13.5%)	Depression (13.5%)	PTSD (10.8%)	Panic Disorder (10.8%)
Math et al. (51)	Tsunami	Depression (21.5%)	Panic Disorder (12%)	PTSD (11.2%)	Anxiety Disorder (5.5%)
Seethalakshmi et al. (32)	Motor Cycle Accident	Depression (30%)	Anxiety (30%)	PTSD (20%)	
Servan-Schreiber et al. (74)	Displacement	PTSD (11.5%)	Depressive Disorder (11.5%)		
Sethi and Bhargava (33)	Suicide Survivors	Major Depressive Disorder (50%)	PTSD (21%)	Conduct Disorder (0.13%)	Panic Disorder (0.04%)
Tichy et al. (27)	Domestic Abuse	PTSD (37%)	Acute PTSD (25%)	Acute Stress Disorder (22%)	
Viswanath et al. (66)	Tsunami	Adjustment Disorder (37%)	Depression (21.5%)	Panic Disorder (12%)	PTSD (11.2%)

#### What Measures Are Taken for Addressing PTSD and PTSS in the Indian Setting?

##### Psychosocial Care

There is a lack of assessment of trauma-specific interventions. While we identified an overwhelming majority of studies to give health assessments and overviews of the problem (n = 38), we found few studies to provide solutions. For the studies that do report some form of intervention, the most commonly mentioned interventions were some form of psychosocial support (n = 8) (25, 41, 44, 47, 52, 62, 63, 65). Referring to the study of Becker (41) investigating the impact of psychosocial care on women survivors of the 2004 tsunami, the intervention consisted of receiving emotional support, learning relaxation techniques, discussing means of improved livelihood, and encouragement to speak about their experiences. Pre- and post-IES scores indicated a significant decrease in PTSD symptoms following the intervention (41).

One study administered the psychosocial support approach in a unique, yet simple way. In their 2008 study on mental health support for tsunami survivors in Tamil Nadu, Vijayakumar and Kumar (65) used befriending as a form of psychosocial support for the bereaved victims. This simple, yet effective approach involves trained volunteers who make themselves available, offering human contact and emotional support to the participants. Neither insight nor advice is given, but the volunteers serve as a platform in which the pros and cons of possible coping mechanisms and their consequences can be explored. Befriending works on the same principles as friendship, in which total availability, confidentiality, unconditional acceptance, and emotional support are key (65). Following this intervention, the team saw a significant decrease in PTSD scores in the intervention group 12 months following the baseline survey as well as a significant difference between the intervention and control group at this same time point.

Other studies that implemented the psychosocial approach have used less comprehensive interventions, such as simple group discussions (52). On studying the psychological impact of the 2004 tsunami on children in the union territory of the Andaman Islands, Math et al. (52) applied simple group discussions that entailed having students discuss the problems they have experienced as a consequence of the disaster and different types of positive and negative coping strategies.

##### Psychotherapy

Few studies mentioned exclusively psychological methods of treating PTSD. In the case study undertaken by Raju (30), a 45-year-old adult male who had witnessed violence as a child underwent Eye Movement Desensitization and Reprocessing (EMDR) psychotherapy along with ego strengthening under hypnosis to treat his PTSD symptoms of intrusive memories of the incident, constant tension, nightmares, inability to connect emotionally in relationships, and general numbness. Starting with a specific image of the trauma and associated negative cognition, the patient underwent desensitization in a series of sessions. Other emerging images were also desensitized as per EMDR, using eye movements. By the end of the desensitization, the patient did not have any disturbance associated with the original image and was reported to be asymptomatic over the monthly follow-up over 8 months (30).

In another intervention study on a population of tsunami survivors, Descilo et al. (45) applied exposure therapy, also known as Traumatic Incident Reduction (TIR) as a means of reducing PTSD. For this intervention, patients are initially flooded with cues associated with the traumatic memories in order to induce a similar state to that during the event. With repetition, the painful effects of the memories are reduced until they are no longer averse. At 6 weeks following the intervention, the mean PTSD scores measured by PCL were reduced by 60% (45). As this intervention was applied in combination with other methods, it cannot be stated that this reduction in PTSD symptoms is exclusively due to the exposure therapy.

##### Alternative Approaches

A few studies described the implementation of alternative approaches to treating PTSD symptoms. Play therapy was described as a highly effective form of treatment for PTSD in children who had experienced sexual abuse. Bhaskaran et al. (25, 26) described a weekly play therapy intervention in which trauma narratives were elicited through art or play material. The intervention focused on the expression of emotions and personal safety skills training (25). Yoga was another non-conventional intervention that was applied to reduce PTSD symptoms and distress among different populations of natural disaster survivors (45, 61, 81).

##### Other Interventions

Other interventions not so commonly mentioned include disaster-specific interventions, which usually consisted of an integrated and holistic approach to care that included first aid, counseling, and various forms of social support (47, 56). Pharmacotherapy was only implemented in two studies (25, 44). In the case study of two pre-school children who had PTSD as a result of sexual abuse (25), pharmacotherapy was implemented in combination with play therapy for the children and psycho-education for the parents. Moderate reduction in PTSD symptoms was reported between 6 and 12 weeks following the events.

##### Cultural Sensitivity of Interventions

An important finding from the analysis of the studies that implemented intervention strategies following a traumatic event was that the integration and adaptation of interventions to fit local context are crucial to their success. In this review, a little over half (10 out of the 17) studies that applied interventions took different steps to make these interventions better suited to fit the local context. Findings reveal that the different steps taken to make these interventions more contextually appropriate focused on three different aspects: i) the needs of the population, ii) the cultural norms of the population, and iii) the behavioral norms of the population.

Starting with needs, a number of interventions by the different studies mentioned were developed specifically to cater to the needs of the population in question (47, 62, 63). In their 2008 study, Jeyanth and Jawahar outline a comprehensive disaster mental health intervention, dubbed the Environment, Livelihood, Infrastructure and Institution (ELII) approach following the 2004 tsunami. In addition to individual psychosocial support, the intervention adopted by the Indian red cross society takes a community-based holistic approach to trauma treatment, where care and rehabilitation goes beyond the mental health of the individual, but also emphasizes a focus on improved livelihoods, environments, infrastructure, and institutions. The ELII approach was designed to be technically appropriate and sensitive to both the cultural and language diversity of the settings in which it is implemented (47). In their comprehensive intervention curriculum, Vijayakumar et al. (64) consulted an interdisciplinary team consisting of local experts, community workers, mental health professionals, volunteers, and community gate keepers in order to identify and prioritize the issues faced by the target population of 65 adolescent tsunami victims. Despite such efforts, however, the intervention was not considered successful in reducing trauma-specific symptoms as it was not targeted specifically for this. Authors express that the intervention module was not adequately validated and tested for use in this population (64).

Several of the intervention studies made efforts to adapt interventions to incorporate some of the different cultural practices that exist in the communities of the target populations (41, 45, 60, 61, 65, 71). In a study on torture in Tibetan refugee survivors, an interdisciplinary rehabilitation program was implemented in which the mental health needs of the victims were taken care of through a collective integrated system that combined both Tibetan traditional medicine and modern allopathic medicine (71). In this way, the authors hoped to achieve a treatment protocol that better fit the wishes and needs of the patients. In the previously described intervention of psychosocial care for women survivors of the 2004 tsunami, Becker (41) incorporated cultural rituals and spirituality into the sessions. Additionally, practical issues were discussed with sensitivity, in keeping with social norms, and care was taken to preserve cultural traditions while maintaining continuity of care (41).

Though not commonly addressed, an important cultural adaptation to trauma-related interventions entails adjustment to better fit societal norms. While exploring the psychological impact of the 2004 tsunami in children, Math et al. (52) applied art therapy to their research subjects as a means to identify patients that were most severely impacted by the event. In this study, the author reported that the decision to apply art therapy as treatment was made because parents often discouraged their children from speaking about the event, even when they wanted to—making it difficult to employ traditional talk therapies in this context. As a way around this, art therapy was introduced as a means to allow the children to express themselves while at the same time respecting the culturally motivated decision of the parents (52).

Despite the fact that over half of the studies with interventions have taken efforts to adapt their protocols to better fit the contexts of their target populations, several studies have implemented interventions without any such modifications, including the psychotherapy interventions of EMDR by Raju (30) and TIR by Descilo et al. (45). In a 2016 longitudinal study investigating the clinical features of childhood sexual abuse (CSA), Bhaskaran et al. followed a sample of 40 children and their families over a period of 3 years, during which a combination of psychotherapy and medication was administered as treatment. During this period, findings revealed that a staggering 70% of families lost to follow-up. Suggested reasons for loss to follow-up mainly stemmed from contextual circumstances that were not considered when the intervention was rolled out, namely, family occupation with legal battles, stigma associated with abuse, lack of knowledge regarding mental health impact of CSA, and long waiting times in the public health care system (26).

## Discussion

The results of this review provide an overview of the many elements that conceptualize PTSD in India. The diversity in types of studies, methods employed, subjects of focus, and outcomes of the 56 studies included only confirm the complexity that is PTSD in the sub-continental context. Though the findings reveal that socio-cultural, political, and geographic contexts have influenced the perceptions and focus of PTSD in India, western-derived conceptualizations of PTSD, particularly the DSM, remain dominant. This eurocentrism is evident in both clinical and research practice, as is evident in the key observations of this study outlined below.

### There Is a Disproportionate Amount of Attention Towards “Big T,” and Non-Interpersonal Traumas and a Lack of Attention Towards “Small T” and Interpersonal Traumas in the Indian Context

With natural disasters and conflict cumulatively making up more than two-thirds of the types of traumatic events studied, the results of the dataset reveal that mainly conventional big T types of traumas and non-interpersonal traumas were studied, such as natural disasters, war, and transportation or man-made accidents. This review revealed that significantly less attention is drawn to small t traumas in the Indian setting—events that do not quite fit the criterion A of PTSD, yet still exceed one’s capacity to cope, such as divorce, financial loss, and bullying (82). In a collectivist society such as India, where there is a general interdependence among people, and an emphasis on group ambitions (81), the breakup of familial structures due to divorce, alienation, or abandonment can have lasting traumatic consequences. Equally salient is the lack of attention towards interpersonal traumas in the Indian context, which literature has repeatedly shown to cause significantly more distress and risk of PTSD in victims (11).

The study of PTSD from the perspective of conventional traumas is perhaps a reflection of an absolutist/etic approach, which according to Vikram Patel (4) has dominated the study of psychiatry in India. The main criticism of an etic approach, in which emphasis is placed on diagnostic criteria most frequently developed in the west, is that these criteria may only be relevant in the context of which they were developed and not necessarily in the context they wish to be applied, such as India (4, 83). In more recent years, a shift in perspective has led to a realization of the importance of sociocultural environments in the manifestations of mental illnesses (84, 85). More effort is needed to understand PTSD from a culturally relativist and emic approach—from the perspective of the population in which it is being studied. In order to get a better understanding of the local burden of trauma, it is imperative to understand what types of events people themselves consider to be traumatic and what symptoms they ascribe to these experiences.

### There Is Less Attention to Particularly Vulnerable Populations Susceptible to Traumatic Exposure and PTSD

In addition to the types of events studied, there is also a disproportionate amount of attention in regards to types of populations studied. While nearly one fourth (24%) of the selected studies focused primarily on children and adolescents, there were significantly fewer studies (7%) whose primary focus were women—an equally vulnerable group. Countless studies exist—including those included in this review—that attest to the fact that women are at a significantly higher risk of PTSD, compared to men or the general population (32, 38, 42). A number of reasons suggest why women are at a higher risk of getting PTSD compared to men, including higher risk of exposure to specific types of traumatic events, average younger age at exposure, their social environment that generally provides fewer supportive resources, and stronger perceptions of threat and loss of control (86). These assumptions also hold true in the Indian context. While women’s roles in Indian society are currently experiencing far-reaching changes from mainly being domestic homemakers to gaining more access to education and increasing involvement in social, economic, and political affairs (27), traditional views are still held in many parts of the country—particularly in rural areas—where the limited freedoms have subjected women to considerable victimization. Studies have indicated that past experiences of childhood abuse and sexual assault increases the susceptibility to PTSD in subsequent traumatic experiences (87, 88). In a nation with an unprecedented number of reports on sexual assault against women, more focus is required into investigating PTSD in this particularly vulnerable population.

### PTSD Screening and Diagnosis Remains Highly Specialized

While there seems to be a wide variety of both professional and non-professional mental health workers responsible for screening and clinical diagnosis of PTSD in India, our findings reveal that the overwhelming majority is made up of psychiatrists. With approximately 3,600 psychiatrists for a population of over 1.2 billion, and facing a serious mental health treatment gap (89), there is increasing need for task-shifting opportunities in the Indian context—transitioning the role of trauma diagnosis and intervention from specialized to non-specialized mental health workers as a strategy for maximizing the effective use of resources available and expanding mental health care access where needed (90).

### Indian Culture Shapes the Construct, Manifestations, and Health Seeking Behaviors in Relation to PTSD

This review has provided evidence that reinforces the notion that culture and society shape many aspects of PTSD and PTSS conceptualization, susceptibility, and recovery. For instance, several studies reported a lack of presentation of avoidance symptoms of PTSD. While this observation is also dependent on the type of trauma experienced, a lack of avoidance symptoms can be partially explained by the collectivist culture within Indian society that fosters an open environment for sharing one’s burdens. In their 2013 study on the prevalence and determinants of PTSS following the 2004 tsunami, Rajkumar et al. credited the absence of avoidance symptoms among tsunami survivors to the cohesive social bonds and religious rituals that facilitated talking about the event and collective mourning in the community (5).

Religious and spiritual beliefs may also influence trauma susceptibility. Research has indicated that locus of control plays a role in susceptibility to PTSD. While an internal locus of control—the belief that the outcomes of events in your life are in your own control—seems to provide a protective role in people subjected to traumatic experiences, an external locus, in which events in one’s life are believed to be controlled by outside forces, tends to instill a sense of helplessness and lack of control in victims of trauma (57). In India where the role of religion and strong beliefs in karma are highly predominant, society is generally characterized by an external locus of control (57, 91).

Of course, culture has implications for what can be considered a traumatic event to begin with. If PTSD is considered a normal reaction to abnormal situations, how does it manifest in situations where violence or other potentially traumatizing events become the norm? In their 2009 study on addressing domestic violence and socio-economic considerations for women in Tamil Nadu, Tichy et al. reveal some of the challenges that arise in these types of situations. In their study, more than half of abused women did not recognize their experience as abuse and this correlated with the inability to recognize abuse as a societal problem, let alone traumatic. This reflects the fact that despite progress in women’s rights and access to societal advancements, the patriarchal societal structure that traditionally defines India remains strongly intact (27). In a society where crimes against women make up 11% of total number of crimes reported under the Indian penal code, and the highest number of crimes reported in any subcategory, female victimization is not uncommon (92).

### Strengths and Limitations of Research

To our knowledge, this is the first known effort to systematically review the peer-reviewed literature on PTSD and PTSS in India. This approach enabled us to methodically search, analyze, and interpret our findings in order to provide the most accurate account of how PTSD and PTSS are conceptualized in this context. This systematic approach has generated valuable data, covering a wide range of information in types of studies, populations studied, and types of traumatic events covered that shed light on the idioms of distress used to describe trauma and PTSD in the Indian context. This review highlights particular trends regarding trauma and PTSD in India, such as what are the most vulnerable populations studied, what types of events, and the most common tools used in identifying trauma. Additionally, it highlights the extent of cultural sensitivity in addressing PTSD and PTSS in the Indian context. At the same time, it illustrates where there are gaps in the research, such as the investigation of everyday traumas that are prevalent in Indian society, including marital disputes or sexual violence against women, neglected populations such as the mentally ill or homeless, and the need for task shifting in PTSD diagnosis and treatment.

There were some limitations. While our systematic approach enabled us to generate the most reliable peer-reviewed and published information regarding PTSD in India, it is questionable whether the language of distress used locally and the true meaning of trauma by those experiencing it is sufficiently explorable using this methodological approach. Additionally, this strategy may have prevented us from identifying non-peer-reviewed studies, including those that could potentially be found in national databases and that may also contribute to the knowledge on PTSD in this context. Additionally, as mentioned earlier, 6 out of the 56 included studies scored insufficient in their methodological assessment. The lowest scores reflected a lack of information for sample selection, a lack of information on methods of analysis, and inadequate reporting of results, such as an indication of variability within the sample population and accounting for confounding factors that may have influenced the findings of the different studies. Due to the limited number of studies relevant to the topic of PTSD in India, all studies identified were included, despite some of their poor methodological qualities. While the quality of these studies included in the review puts into question some of their findings and their interpretations, it illustrates a realistic picture on the type of information that is available and reported on PTSD in India, regardless of their quality.

### Next Steps: Where Do We Go From Here

The findings of this review highlight several important next steps to be taken in both clinical practice and research in the field of trauma. From a clinical perspective, task shifting is a top priority. More recruitment and training of lower level providers of mental health care, particularly in the area of trauma diagnosis and treatment, is needed to help alleviate the current mental health treatment gap experienced in India. Additionally, sensitization to the local idioms of distress used to describe PTSD in the Indian population is required in order to better recognize potential victims of traumatic exposure. From a research perspective, greater emphasis and endorsement needs to be placed on qualitative and mixed-methods approaches to investigating the perceptions and experience of trauma, particularly in vulnerable and notably neglected populations, such as victims of abuse, women, the homeless, and the mentally ill.

## Conclusions

This review puts into focus the complexity in understanding PTSD and PTSS from an Indian perspective. The diversity in classification, measures, and treatment options for PTSD in the Indian context alone reflects the ongoing dilemma in measuring and identifying PTSD and PTSS worldwide. As much as this review has illustrated the diversity in studying PTSD in India, it also reveals the limited scope in terms of types of traumas and types of populations that are studied. There is an obvious need to cater PTSD research to the specific needs of this population and traumatic events considered as such that are outside the traditional western-derived classifications of the DSM. The results of this review only further emphasize the need for gaining local understandings and developing culturally sensitive measures for identifying and addressing PTSD in various populations—an action urgently needed for reducing the so-called global mental health treatment gap.

## Author Contributions

AA and AG systematically searched for the literature, screened articles for inclusion, performed data extraction, and conducted the methodological quality assessments of the included studies. AG drafted and revised all versions of the manuscript. AA and BR reviewed drafts of the manuscript.

## Conflict of Interest Statement

The authors declare that the research was conducted in the absence of any commercial or financial relationships that could be construed as a potential conflict of interest.
